# Supplementary figures and images for: Predictive role of preoperative sarcopenia for long-term survival in rectal cancer patients: A meta-analysis
Source: PLoS One. 2024 May 21;19(5):e0303494. doi: 10.1371/journal.pone.0303494 (PMC11108127; doi:10.1371/journal.pone.0303494)

**Literature search strategies in each database:**

PubMed:


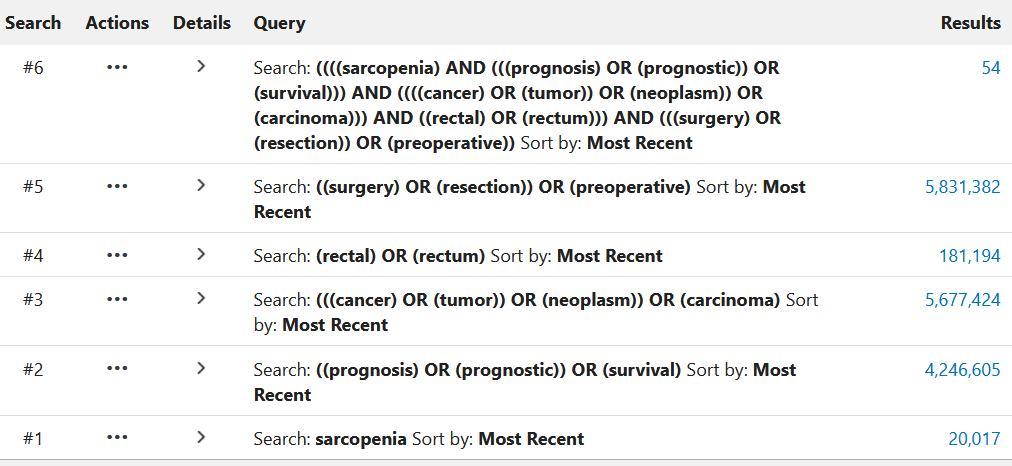


Web of Science:


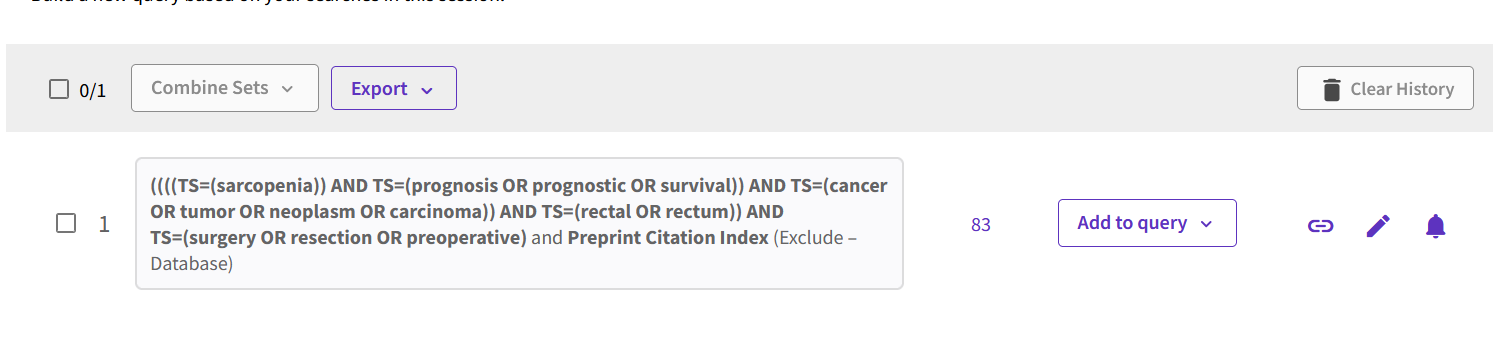


EMBASE:


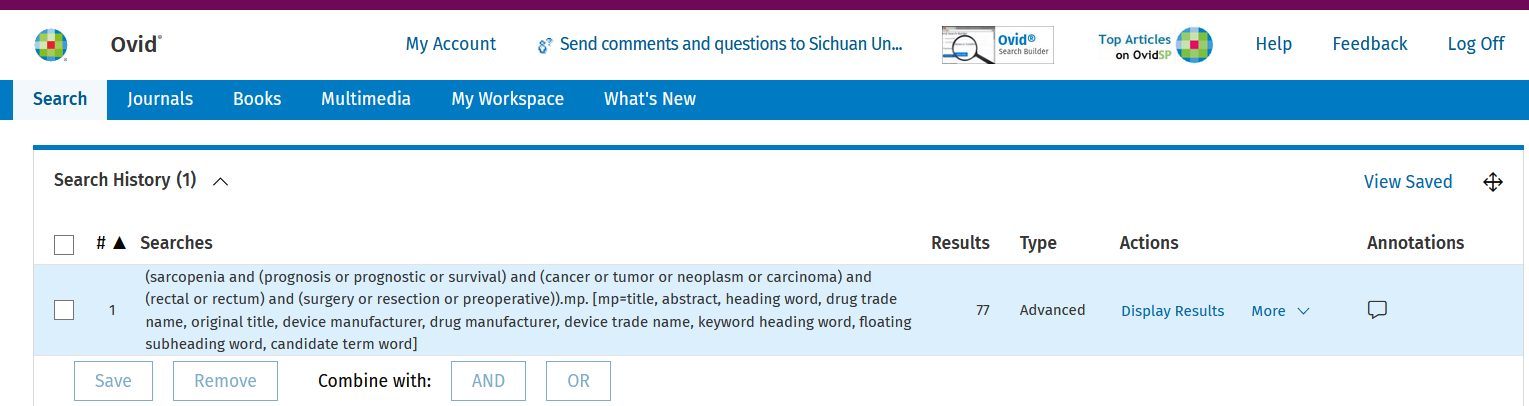

Supplement: S1 File — (DOCX) [file pone.0303494.s001.docx]
