# Supplementary material for: Predictive role of preoperative sarcopenia for long-term survival in rectal cancer patients: A meta-analysis
Source: PLoS One. 2024 May 21;19(5):e0303494. doi: 10.1371/journal.pone.0303494 (PMC11108127; doi:10.1371/journal.pone.0303494)
Supplement: Attachment — Submitted filename: resonse to editor and reviewers.docx [file pone.0303494.s004.docx]

**Response to Reviewer #3:**

I have noticed a minor issue in the Prisma flow diagram (figure 1). The total number of studies included in the meta-analysis has been written as n=25, but I guess it should be n=15 (the authors have written n=15 in the result part, page5/19, line 22). Please take this into consideration.

**Answer:** We are sorry for this mistake. The Figure 1 has been modified and reuploaded.
